# A novel approach for more precise quantification of M-protein using variables derived from immunosubtraction electropherogram and associated biochemistry analytes

**DOI:** 10.11613/BM.2022.030703

**Published:** 2022-08-05

**Authors:** Dragana Šegulja, Danica Matišić, Karmela Barišić, Dunja Rogić

**Affiliations:** 1 Department of Laboratory Diagnostics, University Hospital Centre Zagreb, Zagreb, Croatia; 2 Salzer Polyclinic, Zagreb, Croatia; 3 Faculty of Pharmacy and Biochemistry, University of Zagreb, Zagreb, Croatia

**Keywords:** capillary electrophoresis, M-protein, monoclonal gammopathy, multiple myeloma, serum protein electrophoresis, immunosubtraction electrophoresis

## Abstract

**Introduction:**

Due to limitations in currently used methodologies, the widely acknowledged approach for quantifying M-protein (MP) is not available. If employed as a source of quantitative data, the immunosubtraction electropherogram (IS-EPG), a qualitative analysis of MP, has the potential to overcome known analytical issues. The aim of this study is to explore measured and derived variables obtained from immunosubtraction electropherogram as a tool for quantifying MP and to compare the derived results to currently available methods.

**Materials and methods:**

Measurands were amplitudes of MP and albumin fractions. Assessed derived variables included also immunoglobulin (Ig) G, IgA, IgM and total protein data. Capillary electrophoresis was used for determination of MP (in % of total protein concentration, or concentration of MP in g/L) by perpendicular drop and tangent skimming method.

**Results:**

Passing-Bablok analysis showed the most comparable results in D1Ig and D1nIg variables, and the largest discrepancies in AD1nIg and AD2nIg variables. The background presence had greater impact on D1nIg comparison results than did on D1Ig results. The contribution of albumin fraction data did not improve the comparability of the results. The coefficients of variation of derived variables were lower (maximum 3.1%) than those obtained by densitometric measurements, regardless of MP concentration, polyclonal background, or migration pattern (2.3-37.7%).

**Conclusion:**

The amplitude of MP spike in IS-EPG is an valuable measurand to compute derived variables for quantifying MP. The most comparable results were achieved with the D1Ig variable. Patients with monoclonal gammopathy can benefit from increased precision employing an objective and background independent measurand, especially during longitudinal follow-up.

## Introduction

In the Western world, multiple myeloma is the second most common hematological malignancy (*1*). The International Myeloma Working Group maintains diagnostic and response criteria up to date, recognizing the need for M-protein (MP) quantification (*2*). Although diagnostic and follow-up options in this field have vastly advanced over the last decade, from kappa/lambda ratio measurement to introduction of heavy/light chain assays in laboratory protocols, there is no widely accepted approach for quantification of MP (*3*-*5*). Considering MP is produced by tumor-altered cells, its structure varies, making quantification challenging and explaining the lack of standard material in this field.

One of the most commonly used approach is to measure total concentration of immunoglobulin (Ig) isotype involved in monoclonal synthesis (Ig_invl_) using turbidimetry or nephelometry. This approach frequently yields overstated monoclonal immunoglobulin concentration. The approach which enables estimation of monoclonal fraction is densitometry. This methodology is appropriate if MP is found in the gamma globulin fraction. Factors including a high polyclonal background, the positioning of MP in the beta fraction, and the polimerization of immunoglobulin molecules limit its effectiveness. Additionally, the fact that densitometric measurement can be done using one of two models: tangent skimming or perpendicular drop, both which lack the objectivity, contributes to the variability of results (*6*, *7*).

Immunosubtraction is a fully automated method for characterizing MP. The method utilizes capillary electrophoresis in combination with immunoprecipitation. Antisera are coupled to sepharose beads which alter mobility in an electric field by binding to immunoglobulin molecules and forming immunocomplexes. Comparison of electropherograms (EPGs) before and after immunoprecipitation enables detection and characterization of MP (*8*). In addition, the level of polyclonal background and comigrating beta fraction proteins can be assessed by comparing EPGs. Detected differences could be described by measured and derived variables. The aim of this study was to explore measured and derived variables obtained from immunosubtraction electropherogram (IS-EPG) as a tool for quantifying MP and to compare the derived results to currently available methods.

## Materials and methods

A total of 133 patient samples with monoclonal gammopathy were included in this study. Only serum samples with the requested serum protein electrophoresis were used. There was no additional sample taken particularly for this investigation. The study has been approved by the ethical committee of a tertiary care hospital (8.1-17/55-2).

Total immunoglobulins G, A, and M were determined in all samples, and capillary electrophoresis, immunofixation, and immunosubtraction were conducted. Using Tina-quant reagents, immunoglobulins were determined turbidimetrically on Cobas 6000cee analyser (Roche Diagnostics, Rotkreuz, Switzerland). The Capillarys 2 system (Sebia, Lysses, France) was used for serum protein electrophoresis and immunosubtraction utilizing Capillarys Protein(e) 6 buffer and Capillarys Immunotyping antisera kit (Sebia, Lysses, France). Immunofixation electrophoresis on Hydrasys2Scan with the reagent set Hydragel IF 2/4 was conducted to confirm MPs (Sebia, Lysses, France). All the tests were carried out according to the manufacturer’s instructions. To normalize patient data, the IF/IT Control (Sebia, Lysses, France) sample was employed. M-proteins: IgG lambda, IgA kappa, and IgM lambda were verified in the absence of a polyclonal background in utilized control sample which comprised total protein of 42 g/L, turbidimetrically determined IgA of 2.90, IgG 7.28, IgM 6.28 g/L and densitometrically measured MP IgA of 2.70, IgG 7.40 and IgM 6.10. Precision testing was carried out on serum samples, with four known variable factors in MP quantification taken into account: migration pattern, polyclonal background, MP concentration, and gating method. The measurements are performed in hexaplicate, and two observers were included to inspect variation in gating strategy.

### Calculations

**Figure 1:**
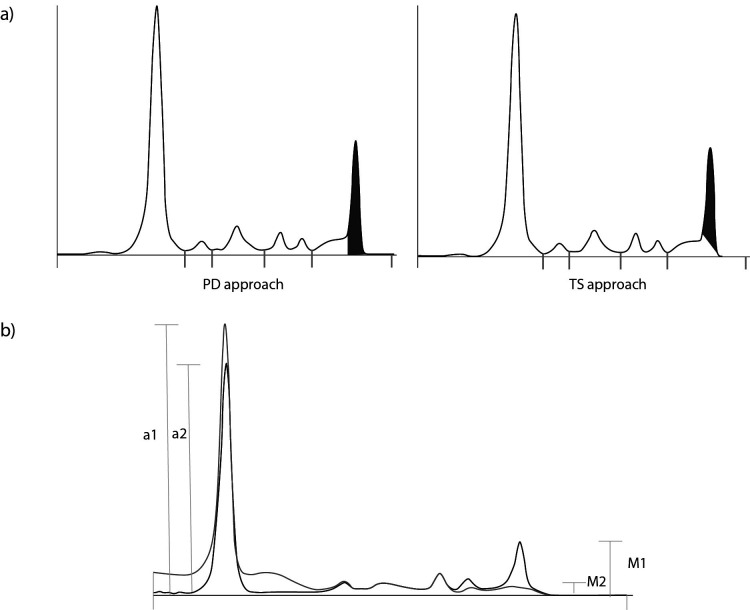
(a) Currently used densitometric approaches to quantify MP are perpendicular drop (PD) and tangent skimming method (TS). (b) Parameters generated from an immunosubtraction electropherogram that were employed in eight evaluated M-protein quantification models. MP – M-protein. M1 - the amplitude of M spike before immunoprecipitation. M2 - the amplitude of the globulin fraction after immunoprecipitation. a2 - the amplitude of albumin fraction before immunoprecipitation. a1 - the amplitude of albumin fraction after immunoprecipitation.

Monoclonal fraction was obtained from standard EPG densitometrically by two mathematical approches. A perpendicular drop (PD) in points where the M-spike meets the polyclonal region, as well as a tangent skimming procedure (TM) that eliminates the polyclonal background and quantifies just the M-spike above given points, are used to determine the area under the curve (Figure 1a). The percentage of the area under the curve attributed to MP and albumin fraction together with the total protein concentration obtained by the biuret method on Cobas 6000cc (Roche Diagnostics, Rotkreuz, Switzerland) were used to compute absolute concentrations.

The amplitude of MP and albumin fraction in IS-EPG (Figure 1b) were described with the measured variables M1, M2, a1 and a2. The derived variables are calculated in MS Excel (Microsoft, Redmond, USA) as follows:

AD - the difference in the amplitude of MP spike before and after immunoprecipitation generated from the IS-EPG, (M_1_ - M_2_);

ADn - the ratio of the AD in patient sample and the AD in the control sample, (M_1_ - M_2_)_p_ / (M_1_ - M_2_)_c_;

AD1nIg – the product of ADn, total protein concentration ratio in patient and control sample (TP_p_ / TP_c_) and total concentration of immunoglobulin isotype involved in monoclonal synthesis in patient sample (Ig_invl_), ((M_1_ - M_2_)_p_ / (M_1_ - M_2_)_c_) x (TP_p_ / TP_c_) x Ig_invl_ (g/L);

AD2nIg – the AD1nIg with added difference in albumin fraction amplitude generated from IS-EPG (a1-a2), ((M_1_ - M_2_)_p_ + (a1 - a2)_p_) / ((M_1_ - M_2_)_c_ + (a1 - a2)_c_) x (TP_p_ / TP_c_) x Ig_invl_ (g/L);

ADnG – the product of ADn and globulin concentration (G), obtained as total protein - albumin fraction, ((M_1_ - M_2_)_p_ / (M_1_ - M_2_)_c_) x G (g/L);

D1Ig – the ratio of AD and MP amplitude before immunoprecipitation generated from IS-EPG multiplied by total concentration of immunoglobulin isotype involved in monoclonal synthesis in patient sample (Ig_invl_), (M_1_ - M_2_) / M_1_) x Ig_invl_ (g/L);

D2Ig – the D1Ig with added albumin fraction data generated from IS-EPG and multiplied by total concentration of immunoglobulin isotype involved in monoclonal synthesis in patient sample (Ig_invl_), ((M_1_ - M_2_) / M_1_ + (a_1_ - a_2_) / a_2_) x Ig_invl_ (g/L);

D1nIg – the ratio of AD and MP amplitude before immunoprecipitation generated from IS-EPG in patient and control sample multiplied by TP_p_ / TP_c_ and by total concentration of immunoglobulin isotype involved in monoclonal synthesis in patient sample, ((M_1_ - M_2_) / M_1_)_p_ / ((M_1_ - M_2_) / M_1_)_c_ x (TP_p_ / TP_c_) x Ig_invl_ (g/L).

### Statistical analysis

All data sets were tested for normality using Kolmogorov-Smirnov test and presented with median and interquartile range (IQR), except IgM data which follow normal distribution and are presented with arithmetic mean and standard deviation. Comparison results are presented by Bland-Altman statistics and Passing-Bablok regression where slope and intercept values are listed with 95% confidence interval (95%CI). The values P < 0.05 were considered statistically significant. All statistics were done by MedCalc statistical software, version 20.023 (MedCalc, Ostend, Belgium).

## Results

**Figure 2:**
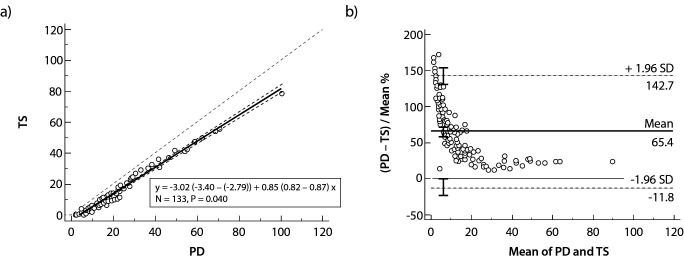
Passing-Bablok regression (a) and a difference plot (b) of results obtained by two densitometric approaches. A positive bias indicates higher values for the PD approach. The 95% confidence intervals for intercept and slope are shown within parentheses. PD - perpendicular drop approach. TS - tangent skimming method.

Monoclonal IgG was detected in 70% of examined samples. Monoclonal IgA was detected in 14% of samples, and monoclonal IgM in 16%. Altogether 30% of detected MPs were lambda type. The majority of IgG MPs were found in the gamma fraction (91%), IgA MPs in the beta fraction (84%), and monoclonal IgM in the gamma fraction (86%). Background, polyclonal or beta fraction proteins were found in 43% of the samples. As shown in Table 1, the study included patient samples with a wide range of total protein, total immunoglobulins, and MP concentrations. The concentration of total immunoglobulin isotype involved in monoclonal synthesis was higher than that determined by densitometrical approaches, with a mean difference of 31.14 (26.25-36.02, P < 0.001) % for PD and 86.85 (78.89-94.81, P < 0.001) % for the TS method. Positive bias for PD results was found in a comparison of two densitometrical methods (Figure 2b), as well as systematic and proportional differences when they were categorized based on background presence (polyclonal background or beta migrating MP) (with background, intercept: - 1.46 (95%CI - 1.88 to - 1.09), slope: 0.57 (95%CI 0.52 to 0.63), P = 0.740; without background, intercept - 2.84 (95%CI - 3.52 to - 2.43), slope 0.85 (95%CI 0.83 to 0.89), P = 0.350).

**Table 1:** Descriptive data of patient samples

	**N**	**Median**	**IQR**	**Min**	**Max**	**P**
Total protein (g/L)	133	76	71-83	53	172	< 0.001
MP IgG (g/L)	93	19.05	14.07-30.98	7.99	103.00	< 0.001
MP IgA (g/L)	19	13.00	7.30-16.72	3.44	59.20	0.002
MP IgM (g/L)	21	13.15*	8.01*	3.79	34.75	> 0.100
PD (g/L)	133	12.15	7.25-20.95	1.90	100.60	< 0.001
TS (g/L)	133	6.60	2.58-14.13	0.20	78.90	< 0.001
MP with background (g/L)^†^	57	7.00	5.10-10.15	1.90	20.60	0.040
MP without background (g/L)^†^	76	19.00	13.10-29.85	5.20	100.60	< 0.001
*IgM data presented with arithmetic mean and standard deviation. ^†^concentrations obtained by PD approach. MP - M-protein. IgG - immunoglobulin G. IgA - immunoglobulin A. IgM - immunoglobulin M. PD - Perpendicular drop approach. TS - tangent skimming approach. IQR - interquartile range. Min - lowest value. Max - highest value.						

**Figure 3:**
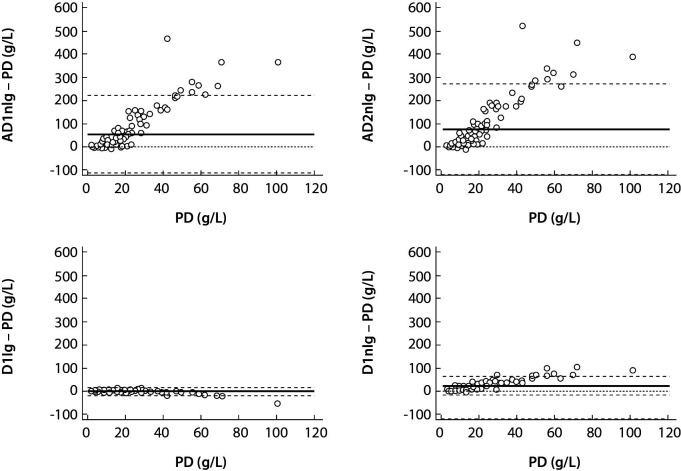
Bland-Altman data comparison graphs show differences for AD1nIg, AD2nIg, D1Ig, and D1nIg variables in regard to the PD densitometric approach. D1Ig, and D1nIg data showed the lowest difference in comparison to PD approach results. In normalized results is evident increasing tendency in difference with increase of MP concentration. Solid line (mean) – mean difference; dashed lines (SD) – standard deviation. AD1nIg – normalized, includes MP amplitude data, total protein and concentration of total immunoglobulin isotype involved in monoclonal synthesis (Ig_invl_). AD2nIg – AD1nIg with contribution of albumin data. D1Ig – includes proportion change in MP amplitude data before and after immunoprecipitation, as well as concentration of total immunoglobulin isotype involved in monoclonal synthesis (Ig_invl_). D1nIg – normalized D1Ig. PD - perpendicular drop approach. MP – M-protein.

**Figure 4:**
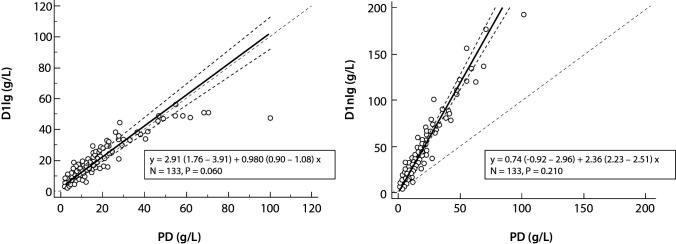
Passing-Bablok regression analysis of D1Ig and D1nIg values in comparison to PD results; proportional differences were observed in D1nIg results and systematic differences in D1Ig results. The 95% confidence intervals for intercept and slope are shown within parentheses. D1Ig – includes proportion change in MP amplitude data before and after immunoprecipitation, as well as concentration of total immunoglobulin isotype involved in monoclonal synthesis (Ig_invl_). D1nIg – normalized D1Ig variable. PD - perpendicular drop approach. MP – M-protein.

Derived variables with included concentration of total immunoglobulin isotype involved in monoclonal synthesis - AD1nIg, AD2nIg, D1Ig, and D1nIg – achieved comparable results with both densitometrical approaches. The variables with applied normalization, AD1nIg, AD2nIg and D1nIg, revealed a clear tendency of increasing difference with increase of MP concentration, especially above 20 g/L assessed by PD method (Figure 3). Variables D1Ig and D1nIg achieved the most comparable results (Figure 4). The AD1nIg results showed a significant deviation from linearity (P < 0.010), while the AD2nIg results (intercept - 33.18 (95%CI - 40.59 to - 26.61), slope 7.03 (95%CI 6.50 to 7.53); P = 0.210) indicated systematic and proportional differences.

When data were classified into two groups based on the presence of background (polyclonal background or beta migrating MP) and compared to densitometrical results, the most comparable results were also noted in D1Ig and D1nIg variables. Only samples with MP concentrations less than 20g/L, determined by PD, were included in the regression analysis of normalized data (Table 2). No systematic or proportional differences were observed in D1Ig results comparing to PD approach in group with background, while there were moderate systematic differences in group without background. The implementation of a 15 g/L MP concentration cut-off had no effect on the outcome, while groups with concentrations less than 10 mg/L were too small to conduct regression analysis.

**Table 2:** Regression analysis of derived variables data in relation to current densitometric methods and the presence of background

	**Samples with MP and polyclonal background in gamma fraction or with beta migrating MP (N = 57)**			Samples with MP in gamma fraction without polyclonal background (N = 76)*		
	**Intercept**	**Slope**	**P**	**Intercept**	**Slope**	**P**
	**95%Cl**	**95%Cl**		**95%Cl**	**95%Cl**	
**Perpendicular drop approach**						
AD1nIg	3.73	0.23	P = 0.100	- 45.42	5.84	P = 0.530
	2.88 to 4.24	0.17 to 0.33		- 72.46 to - 27.09	4.59 to 7.58	
AD2nIg	- 18.01	5.47	P = 0.520	- 8.07	0.12	P = 0.530
	- 30.12 to - 10.80	4.70 to 7.37		6.11 to 10.00	0.09 to 0.16	
D1Ig	2.24	1.19	P = 0.190	3.48	0.94	P = 0.350
	- 2.12 to 4.38	0.89 to 1.65		1.00 to 5.11	0.84 to 1.06	
D1nIg	0.03	2.81	P = 0.100	- 11.17	3.19	P = 0.150
	- 5.46 to 4.18	2.35 to 3.44		- 23.04 to - 5.66	2.68 to 4.03	
**Tangent skimming approach**						
AD1nIg	- 5.06	7.78	P = 0.320	- 24.14	6.73	P = 0.970
	- 12.05 to - 0.89	5.50 to 11.65		- 42.45 to - 12.49	5.33 to 8.99	
AD2nIg	- 8.24	11.39	P = 0.930	- 40.21	10.01	P = 0.530
	- 15.78 to - 0.04	8.49 to 15.20		- 75.54 to - 22.66	7.79 to 14.06	
D1Ig	3.88	2.40	P = 0.190	6.73	1.09	P = 0.080
	0.71 to 5.49	1.67 to 3.58		4.74 to 7.78	0.96 to 1.25	
D1nIg	4.97	5.50	P = 0.520	- 1.16	4.02	P = 0.530
	- 0.90 to 7.70	4.17 to 7.88		- 10.61 to 3.55	3.18 to 5.24	
In all 57 samples with MP and polyclonal background in gamma fraction or with beta migrating MP, as well as in 40/76 samples with MP in the gamma fraction without polyclonal background, MP concentrations were less than 20g/L. The slope and intercept are listed with 95% confidence interval (CI). *In comparison of normalized variables are included only samples with MP concentration < 20g/L obtained by PD approach (N = 40). MP - M-protein. PD - Perpendicular drop approach. AD1nIg – normalized, includes MP amplitude data, total protein and concentration of total immunoglobulin isotype involved in monoclonal synthesis (Ig_invl_). AD2nIg – AD1nIg with contribution of albumin data. D1Ig – includes proportion change in MP amplitude data before and after immunoprecipitation, as well as concentration of total immunoglobulin isotype involved in monoclonal synthesis (Ig_invl_). D1nIg – normalized D1Ig.						

Comparing to TS approach, none of the derived variables achieved regression without systematic or proportional differences, regardless of background presence.

The highest CVs were noted in TS results. Regardless of MP concentration, polyclonal background, or migration pattern, CVs of derived variables were lower (maximum 3.1%) than those obtained by densitometric measurements (highest 37.7%) (Table 3). Among all evaluated approaches the lowest CVs were observed in the patient sample with MP located in gamma fraction (MP 16.6 g/L by PD; 12.5 g/L by TS), in absence of polyclonal background. In contrast to densitometric approaches, there was no loss of precision in patient samples with low MP concentrations and a pronounced polyclonal background.

**Table 3:** Testing of analytical precision by taking into account four known variable factors in MP quantification: migration pattern, polyclonal gamma globulin background, MP concentration and gating method

**CV%**	**PD**	**TS**	**AD1nIg**	**AD2nIg**	**D1Ig**	**D1nIg**
MP in beta fraction in concentration of 10.9 g/L by PD approach (4.5 g/L by TS approach)	8.3	27.8	2.7	2.4	1.3	1.1
MP in gamma fraction in concentration of 16.6 g/L by PD approach (12.5 g/L by TS approach) without polyclonal background	2.3	8.5	1.0	1.1	0.5	0.6
MP in gamma fraction in concentration of 18.4 g/L by PD approach (11.8 g/L by TS approach) with polyclonal background	6.6	16.8	2.5	2.8	3.1	2.6
MP in gamma fraction in low concentration of 4.1 g/L by PD approach (0.8 g/L by TS approach) with a pronounced polyclonal background	14.4	37.7	2.2	2.3	2.0	2.2
To inspect variation in gating strategy two observers were included. Precision testing results of currently utilized PD and TS approaches and the studied model revealed lower coefficients of variations (CVs) in the studied approach. MP - M-protein. PD – perpendicular approach. TS – tangent skimming approach. AD1nIg – normalized, includes MP amplitude data, total protein and concentration of total immunoglobulin isotype involved in monoclonal synthesis (Iginvl). AD2nIg – AD1nIg with contribution of albumin data. D1Ig – includes proportion change in MP amplitude data before and after immunoprecipitation, as well as concentration of total immunoglobulin isotype involved in monoclonal synthesis (Iginvl). D1nIg – normalized D1Ig.						

## Discussion

Our findings confirm that total immunoglobulin isotype concentration, frequently used as a measure of MP concentration, overestimates the concentration of MP (*9*). In studied group of patients it has been demonstrated that densitometric methods for MP quantification cannot be used interchangeably, regardless of the presence of a background. Similar discrepancies were observed by Schild who proposed TS approach, while Keren and Schroeder described corrected perpendicular drop in order to improve PD approach (*6*, *10*). Possible irregularities in EPG that are not related to MP, as well as the findings of an international survey on laboratory practice regarding monoclonal gammopathies which show that many laboratories still assume the identified spike in EPG is MP without typing the protein, are further reasons why densitometrical approaches for quantification of MP are not most appropriate (*11*-*13*).

In our group of patients the strongest correlation results were achieved using derived variables which included total immunoglobulin isotype involved in monoclonal synthesis. The impact of possible overestimation of total immunoglobulin concentration due to immune reagents reacting differently to specific monoclonal protein amino acid sequences is not eliminated in the proposed models, but it is minimized by visualization of MP in IS-EPG and taking total protein concentration into account (*9*). The D1Ig results, which were not normalized, showed that the D1Ig variable and the PD approach can be used interchangeably in a group with the detected background. The assumption that employed and only accessible material containing three isotypes of MPs with no polyclonal background will improve the quantification model, was not supported with conducted comparisons. We hypothesized that the low concentration of MPs in the used material was the main limitation of normalized variables. Despite the fact that immunoprecitated complexes migrate in the albumin and prealbumin fractions, the albumin fraction data had no influence on the explored quantification strategy.

Previously, the quantitative immunosubtraction approach was described by Schroeder *et al.* (*14*). They employed additional software to export IS-EPG results, and samples from only three patients were analyzed. Bergon and Miravalles developed a different strategy, estimating MP indirectly using polyclonal immunoglobulin heavy chain/light chain equivalency factors measured experimentally (*15*).

Immunosubtraction electrophoresis provides quantitative data which improve MP quantification with increase in precision, especially in samples with detected background and low MP concentration.

The fact that laboratory scientists have been occupied with MP quantification in the previous decade is substantiated by a conducted international multicenter study whose results point to four variable factors in MP quantification: gamma globulin background, migration pattern, MP concentration and gating method (*16*).

According to previously published papers, PD is the preferred approach for quantifying MP in gamma region, and total immunoglobulin concentration involved in monoclonal synthesis or the TS method are preferred methods when MP is in non-gamma regions (*17*). Substantial imprecision in TS results was noted in the sample with beta globulin migrating MP (27.8%), which was attributed to subjectivity in gating across observers. Our results also show that background which was detected in 43% of our results, is not an uncommon occurrence and alters the relevance of using a universal approach for MP quantification, has no or moderate impact on D1Ig and D1nIg variables. The precision of total protein and immunoglobulins is unlikely to have an impact on the precision of computed variables, as both were verified and found to be acceptable during analytical evaluation mandatory by ISO 15189.

The well defined points in IS-EPG, baseline and spike amplitude, are used in this study to avoid lack of objectivity which is a known weakness of densitometrical approaches due to the possibility of subjective MP spike demarcation. Therefore the possibility of a biased densitometrical approach being used interchangeably was eliminated, minimizing inconsistency in findings and the risk of inadequate therapy monitoring. Nowadays, commercially available immunosubtraction systems are limited on detection of IgG, IgA and IgM isotypes. The assessed strategy would be unable to quantify IgD MP, which is extremely rare, accounting for just 1.8 to 1.2% of all multiple myeloma cases, and IgE gammopathy (*18*). In addition, similar as in currently available approaches, the polimerization of immunoglobulin molecules which is presented as two spikes in EPG, could limit the effectiveness of the proposed model.

In conclusion, we described and studied a novel approach for objective quantification of MP by deriving parameters obtained from IS-EPG in combination with relevant biochemistry analytes. For the first time, an approach is presented that encompasses the idea of normalizing/calibrating IS-EPG data. The amplitude of the MP spike in IS-EPG before and after immunosubtraction has been identified and verified as a useful measurand for estimating MP concentration. The D1Ig variable produced the most comparable results in relation to PD approach.

According to our results, most commonly used PD approach can be used interchangeably with the D1Ig variable in group of patients with the present background, where employment of D1Ig increases MP quantification precision. Studied strategy can improve the harmonization of MP findings and follow-up in monoclonal gammopathy patients.
